# Changes in the consumption of antiepileptics and psychotropic medicines after starting low dose naltrexone: A nation-wide register-based controlled before-after study

**DOI:** 10.1038/s41598-019-51569-z

**Published:** 2019-10-21

**Authors:** Guttorm Raknes, Lars Småbrekke

**Affiliations:** 1 Regional Medicines Information and Pharmacovigilance Centre (RELIS), University Hospital of North Norway, Tromsø, Norway; 2 Raknes Research, Ulset, Norway; 3 Department of Pharmacy, Faculty of Health Sciences, UiT - The Arctic University of Norway, Tromsø, Norway

**Keywords:** Therapeutics, Health care

## Abstract

In this controlled before-after study based on data from the Norwegian Prescription Database, we examine whether starting off-label use of Low Dose Naltrexone (LDN) is followed by changes in the consumption of psychotropic medicines including antiepileptics. Patients that collected LDN for the first time in 2013 (N = 11247) were included and stratified into three groups based on LDN exposure. We compared differences in means of cumulative number of defined daily doses (DDD) as well as changes in the number of users one year before and one year after starting LDN. There was a dose-response association between increasing LDN exposure and reductions in the number of users of antiepileptics, antipsychotics and antidepressants. There were significant difference-in-differences in DDDs between the groups with the lowest and highest LDN exposure of antipsychotics (1.4 DDD, 95% CI 0.4 to 2.3, p = 0.007), and in number of users of antiepileptics (3.1% points, 95% CI 1.6% to 4.6%, p < 0.001), antipsychotics (2.1% points, 95% CI 1.2% to 3%, p < 0.001), and antidepressants (2.8% points, 95% CI 1.1% to 4.4%, p = 0.001). The findings show an association between the initiation of persistent LDN use and reduced consumption of several psychotropic medicines and antiepileptics. Beneficial effects of LDN in the treatment of psychiatric diseases cannot be ruled out.

## Introduction

Off-label use of low dose naltrexone (LDN), typically <5 mg/day, is by some patients, doctors and researchers believed to have therapeutic effects in several conditions such as autoimmune diseases^1^ and chronic pain. LDN could be a cheap and safe^2^ alternative to standard treatments, but the evidence of effect is limited. The main use of naltrexone within psychiatry has so far been as treatment of opioid and alcohol addiction^3^, but a recent report suggests that LDN may be helpful in other psychiatric conditions^4^. Some of the beneficial effects in patients with autoimmune diseases have been improvements in quality of life^5,6^ and improvements in symptoms associated with depression, such as sleep and mood^7^. In a study on major depressive disorder where the authors randomized 12 patients to naltrexone 1 mg/day or placebo, there were borderline significant improvements in some clinical depression measures^4^. These results are part of the documentation in a patent application on the use of LDN in depression and other mental health issues^8^. In a randomized study on Gulf war illness (N = 37), there were improvements in general disability, depression and confusion in users of LDN^9^. There have been promising results in a small, randomized study in fibromyalgia, a condition where psychiatric symptoms may be prominent^6^.

A sudden and large increase in LDN consumption in Norway during 2013^10^ has enabled nationwide quasi-experimental register studies on change in medicine use associated to initiation of LDN use. Previously we have demonstrated an association between starting LDN and changes in medication for inflammatory bowel disease^11^ and rheumatoid and seropositive arthritis^12^, but not in multiple sclerosis^13^. We have shown almost halving of the opioid consumption among persistent LDN users^14^. If there are beneficial effects of LDN in psychiatric disease, it is plausible that this will be reflected in the consumption of medicines. We aimed to test the hypothesis that initiation of LDN use is associated with reductions in the dispensing of psychotropic and antiepileptic medicines.

## Materials and Methods

### Study design and setting

We performed a pharmacoepidemiological nationwide quasi-experimental controlled before-after study using data from the Norwegian Prescription Database (NorPD). NorPD contains individual data on all prescriptions dispensed since 2004 to the entire Norwegian population living outside hospitals. Details on NorPD are published previously^15^. Each prescription in NorPD contains a unique pseudonym for the personal identifier, demographic data on patient and prescriber, the specialty of the prescriber, the Anatomical Therapeutic Chemical classification (ATC) code and the amount of medicine in defined daily doses (DDD), date of dispensing, and location of the dispensing pharmacy^16^. It is possible to follow dispensing to individuals on both reimbursed and non-reimbursed prescriptions. The Norwegian Institute of Public Health hosts the database^17^. We used the following variables in this study: personal identifier, age and sex for the patient, ATC code and product identifying number for all prescribed medicines, date of dispensing, and dispensed volume in DDDs. For a fee, NorPD provided a data file according to their data access procedures.

### Participants

We included all Norwegian residents with at least one LDN prescription in 2013 according to NorPD. The date of the first dispense is the index date, and we recorded all dispensed prescriptions on psychotropic and antiepileptic medicines 365 days before the index date, and from the index date +364 days.

We stratified the patients into three subgroups depending on the number of collected LDN prescriptions after the index date. Patients were included in the persistent user subgroup (LDN × 4+) if they collected four or more LDN prescriptions. The other subgroups were one-time users (LDN × 1), and intermediate LDN users (LDN × 2–3). The patients served as their own controls (before data) and between groups that reflect LDN exposure. The LDN × 1, who likely used LDN for a short time, served as a control group compared to the LDN × 4+ group. The LDN × 2–3 group enabled dose-response comparisons.

The total observation time was 2 years for all participants. The first observation date was theoretically January 1, 2012, and the last observation date was December 31, 2014.

### Outcome variables

Main outcome was difference between the cumulative collected amounts of DDDs and number of users on antiepileptics (ATC N03A), antipsychotics (N05A), anxiolytics (N05B), hypnotics (N05C), antidepressants (N06A) and psychostimulants (N06B) during the 365 days preceding the index date throughout the 364 days succeeding the first LDN dispense. We expressed the difference for each patient by subtracting the number of collected DDDs within all ATC groups the year following the first LDN dispense from the number of DDDs in the year preceding the first LDN dispense.

As secondary outcome, we used equivalent changes in DDD and number of users of some subgroups: Tricyclic antidepressants (N06AA), Selective serotonin inhibitors (N06AB), other antidepressants (N06AX), benzodiazepines (N05BA, N05CD, N03AE01) and z hypnotics (benzodiazepine like) (N05CF), gabapentin and pregabalin (N03AX12, N03AX16), lithium (N05AN) and lamotrigine (N05AX09).

### Statistical methods

The number of patients in NorPD collecting a prescription on LDN in 2013 determined the study size. We used SPSS 25 and Excel 2013 for data analysis, and analyzed all data on an individual level. We used a pairwise two-sided *t*-test to determine the significance of mean changes in the sum of the DDDs per patient in each group for all examined medicines, and calculated 95% confidence intervals (CI) for difference of means. Change in the number of users was expressed as the proportion (% points) of each cohort, together with the 95% CI for the difference of proportion (in % points)^18^. Difference-in-difference of DDDs and proportion of users with 95% CI were calculated. The graphical representation of the cumulative difference in dispensing between the groups with the highest and the lowest LDN exposure (LDN × 4+–LDN × 1) before and after the index date is based on dispensing data.

### Ethics statement

The Regional Committee for Medical and Health Research Ethics of Northern Norway reviewed the study protocol. Due to the encrypted data, the committee concluded that disclosure was not mandatory. The local privacy ombudsman for research at the University Hospital of Northern Norway approved the project. Consent from individual patients is by law not required for research based on NorPD.

## Results

### Patients

**Figure 1:**
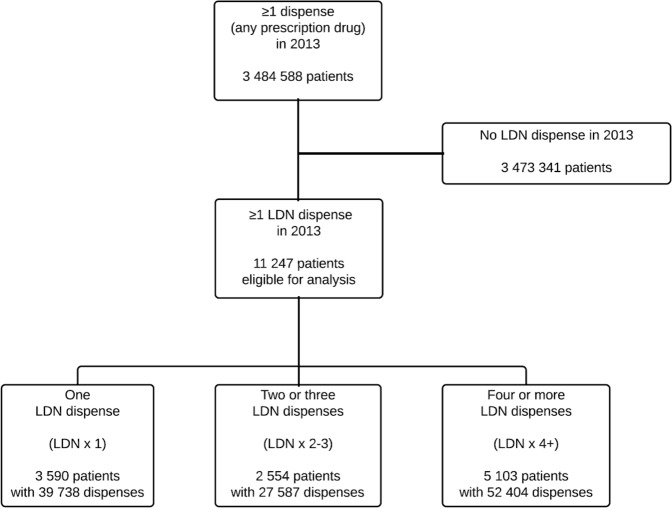
Flow diagram of patient inclusion.

The inclusion of patients and dispenses of outcome medicines is shown in Fig. 1. In total, 11247 patients were included, and the observation period before and after index date constituted 69928 patient-months. The analyses on the examined medicines include 57951 prescriptions dispensed before and 61778 prescriptions dispensed after the index date.

### Descriptive data

Table 1 displays baseline data of the three subgroups. There were three percentage points more women in the LDN × 4+ group, and the mean age was approximately one year higher in this group. There were no missing data on age or sex among the included. The numbers of dispenses of all outcome medicines were comparable in all groups.

**Table 1:** Baseline data.

	LDN × 1		LDN × 2–3		LDN × 4+	
N (%)	3590	(31.9)	2554	(22.7)	5103	(45.4)
Female (%)	2587	(72.1)	1836	(71.9)	3821	(74.9)
Age (SD)	50.6	(12.1)	50.8	(13.6)	51.7	(12.0)
Number of dispenses one year before LDN (outcome medicines) (SD)	5.3	(10.6)	5.1	(12.5)	5.1	(11.3)
Number of users one year before (outcome medicines) (%)	2 127	(59.2)	1 482	(58.0)	2 865	(56.1)
N total	11247					

### Outcome data

**Figure 2:**
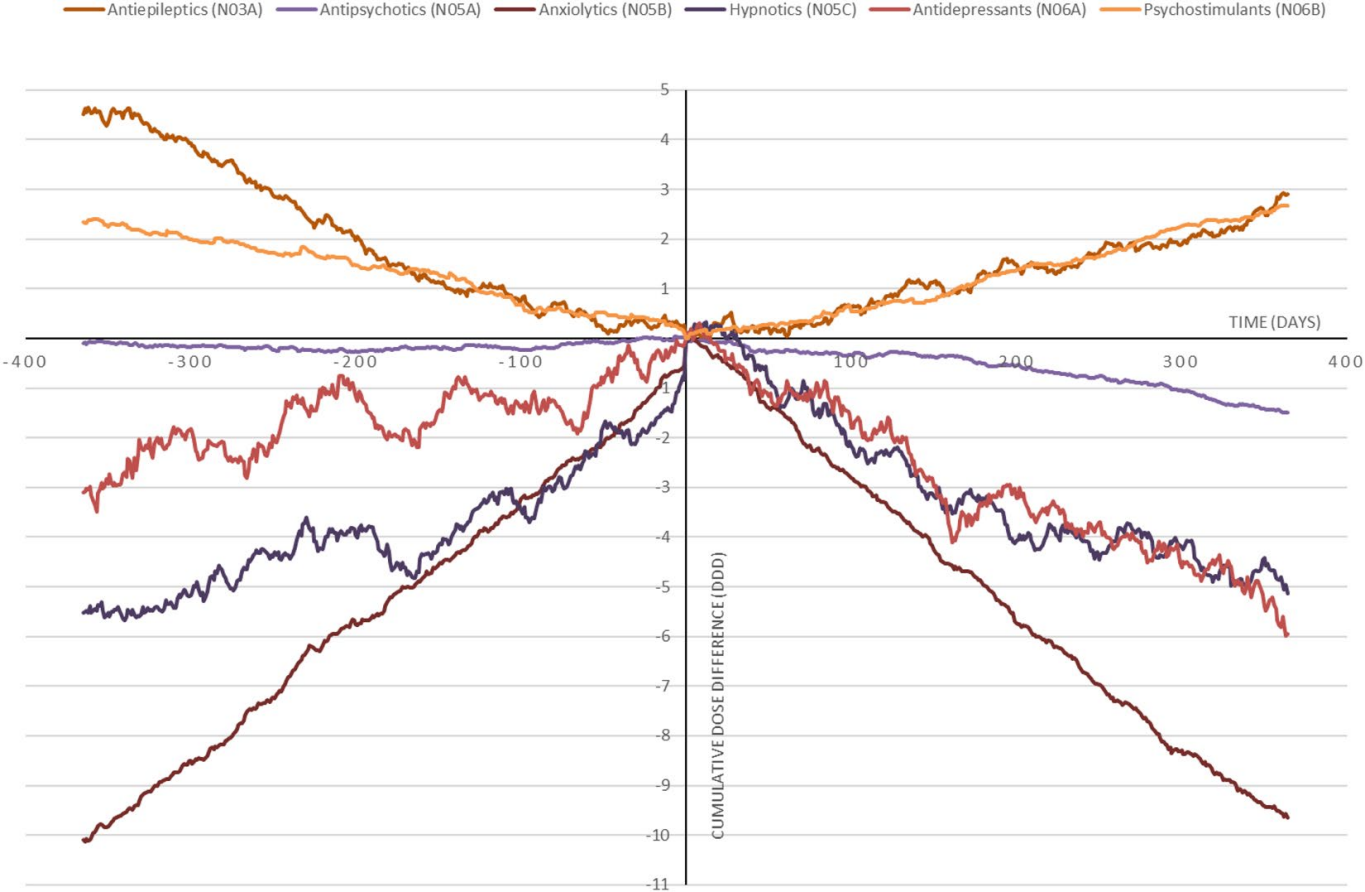
Cumulative difference in dispensed defined daily doses (DDD) of psychotropics and antiepileptics. The curves display difference in DDD between persistent LDN users (LDN × 4+, N = 5103) and patients that collected LDN only once (LDN x1, N = 3590), (LDN × 4+ minus LDN x1). Cumulative differences relative to Index date (Time = 0). Cumulative dose difference >0 means more dispensing to persistent LDN users than to LDN x1. Asymmetry of curve across index date means difference-in-difference before and after starting LDN, this was statistically significant for antipsychotics only. LDN: Low dose naltrexone.

Mean number of dispensed DDDs before and after the first LDN prescription are summarized in Table 2, and changes in the number of users are presented in Table 3. The cumulative differences between LDN × 1 and LDN × 4+ in dispensed DDDs of the main outcomes before and after index date are shown in Fig. 2. Secondary outcomes are presented in Table 4 (DDDs) and Table 5 (number of users).

**Table 2:** Average cumulative dose of main epileptics and psychotropic medicine groups.

Medicine group (ATC code)		Dispensed medicines (DDD)		Difference (DDD)		p
		Before	After	Mean	95% CI	
All examined medicines	LDN × 1	225.8	239.6	13.7	(6.4 to 21.1)	<0.001
	LDN × 2–3	223.2	236.6	13.4	(4.2 to 22.7)	0.004
	LDN × 4+	221.8	224.5	2.7	(−3.0 to 8.5)	0.355
Antiepileptics (N03A)	LDN × 1	38.5	42.9	4.4	(0.6 to 8.2)	0.025
	LDN × 2–3	37.3	40.5	3.2	(−1.9 to 8.3)	0.216
	LDN × 4+	49.2	48.8	−0.4	(−3.4 to 2.6)	0.800
Antipsychotics (N05A)	LDN × 1	3.4	4.4	1.1	(0.2 to 1.9)	0.014
	LDN × 2–3	3.1	4.0	0.9	(−0.1 to 1.9)	0.082
	LDN × 4+	3.3	2.9	−0.3	(−0.7 to 0.1)	0.149
Anxiolytics (N05B)	LDN × 1	26.0	27.8	1.8	(0.1 to 3.5)	0.044
	LDN × 2–3	22.0	23.3	1.3	(−0.6 to 3.2)	0.173
	LDN × 4+	16.4	17.6	1.1	(0.2 to 2.1)	0.019
Hypnotics and sedatives (N05C)	LDN × 1	76.1	81.7	5.6	(2.3 to 9.0)	0.001
	LDN × 2–3	71.5	80.9	9.4	(4.1 to 14.8)	0.001
	LDN × 4+	71.4	75.8	4.4	(1.2 to 7.5)	0.006
Antidepressants (N06A)	LDN × 1	78.1	79.4	1.4	(−2.8 to 5.5)	0.519
	LDN × 2–3	83.5	82.4	−1.1	(−5.6 to 3.4)	0.643
	LDN × 4+	75.2	73.2	−2.0	(−5.2 to 1.2)	0.212
Psychostimulants (N06B)	LDN × 1	3.8	3.3	−0.5	(−1.6 to 0.6)	0.393
	LDN × 2–3	5.8	5.4	−0.4	(−2.2 to 1.4)	0.689
	LDN × 4+	6.2	6.1	−0.1	(−1.1 to 1.0)	0.918

Dispensed to patients one year before and after the first dispense of LDN. Three patient groups based on the number of LDN dispenses: LDN × 1 (*N* = 3590) collected LDN once, LDN × 2–3 (*N* = 2554) two or three times and LDN × 4 + (*N* = 5103) four or more times. LDN: Low dose naltrexone. DDD: Defined daily dose. ATC: Anatomical therapeutic chemical classification.

**Table 3:** Number of users of main epileptics and psychotropic medicine groups.

Medicine group (ATC code		Number of users				Difference		p
		Before		After				
		N	%	N	%	% points	95%CI	
Any examined medicine	LDN × 1	2127	(59.2)	2147	(59.8)	0.6	(−0.9 to 2.0)	0.442
	LDN × 2–3	1482	(58.0)	1525	(59.7)	1.7	(0.0 to 3.4)	0.053
	LDN × 4+	2865	(56.1)	2698	(52.9)	−3.3	(−4.4 to −2.1)	<0.001
Antiepileptics (N03A)	LDN × 1	546	(15.2)	599	(16.7)	1.5	(0.3 to 2.7)	0.015
	LDN × 2–3	360	(14.1)	410	(16.1)	2.0	(0.5 to 3.4)	0.008
	LDN × 4+	807	(15.8)	722	(14.1)	−1.7	(−2.6 to −0.7)	<0.001
Antipsychotics (N05A)	LDN × 1	186	(5.2)	232	(6.5)	1.3	(0.5 to 2.0)	0.001
	LDN × 2–3	137	(5.4)	133	(5.2)	−0.2	(−1.0 to 0.7)	0.710
	LDN × 4+	239	(4.7)	199	(3.9)	−0.8	(−1.3 to −0.3)	0.002
Anxiolytics (N02B)	LDN × 1	829	(23.1)	856	(23.8)	0.8	(−0.4 to 1.9)	0.209
	LDN × 2–3	522	(20.4)	533	(20.9)	0.4	(−0.9 to 1.7)	0.519
	LDN × 4+	912	(17.9)	899	(17.6)	−0.3	(−1.1 to 0.6)	0.573
Hypnotics and sedatives (N05C)	LDN × 1	1143	(31.8)	1173	(32.7)	0.8	(−0.4 to 2.1)	0.176
	LDN × 2–3	779	(30.5)	834	(32.7)	2.2	(0.7 to 3.6)	0.003
	LDN × 4+	1480	(29.0)	1456	(28.5)	−0.5	(−1.4 to 0.4)	0.311
Antidepressants (N06A)	LDN × 1	1045	(29.1)	1039	(28.9)	−0.2	(−1.5 to 1.1)	0.803
	LDN × 2–3	748	(29.3)	730	(28.6)	−0.7	(−2.2 to 0.8)	0.356
	LDN × 4+	1362	(26.7)	1213	(23.8)	−2.9	(−3.9 to −2.0)	<0.001
Psychostimulants (N06B)	LDN × 1	31	(0.9)	41	(1.1)	0.3	(0.0 to 0.6)	0.059
	LDN × 2–3	31	(1.2)	31	(1.2)	0.0	(−0.4 to 0.4)	1.000
	LDN × 4+	74	(1.5)	71	(1.4)	−0.1	(−0.2 to 0.1)	0.467

Dispensed to patients one year before and after the first dispense of LDN. Three patient groups based on the number of LDN dispenses: LDN × 1 (*N* = 3590) collected LDN once, LDN × 2–3 (*N* = 2554) two or three times and LDN × 4 + (*N* = 5103) four or more times. LDN: Low dose naltrexone. ATC: Anatomical therapeutic chemical classification.

**Table 4:** Average cumulative dose of medicines in ATC subgroups (secondary outcomes).

Medicine group (ATC code)		Dispensed medicines (DDD)		Difference (DDD)		p
		Before	After	Mean	95% CI difference	
Benzodiazepines (N05BA + N05CD + N03AE01)	LDN × 1	60.8	62.9	2.1	(−2.2 to 6.4)	0.340
	LDN × 2–3	51.2	51.6	0.4	(−5.1 to 6.0)	0.885
	LDN × 4+	40.0	43.4	3.4	(0.8 to 6.0)	0.011
Z hypnotics (N05CF)	LDN × 1	115.4	124.9	9.5	(3.6 to 15.3)	0.002
	LDN × 2–3	108.6	123.9	15.3	(6.7 to 23.9)	<0.001
	LDN × 4+	102.0	110.2	8.2	(4.1 to 12.2)	<0.001
Tricyclic antidepressants (N06AA)	LDN × 1	9.4	9.5	0.1	(−0.9 to 1.1)	0.847
	LDN × 2–3	9.9	10.1	0.2	(−0.9 to 1.2)	0.773
	LDN × 4+	10.8	10.0	−0.9	(−1.6 to −0.2)	0.017
SSRI (N06AB)	LDN × 1	48.1	48.0	−0.1	(−3.4 to 3.2)	0.950
	LDN × 2–3	53.2	51.3	−1.9	(−5.9 to 2.1)	0.358
	LDN × 4+	43.8	43.7	−0.1	(−2.4 to 2.2)	0.962
Other antidepressants (N06AX)	LDN × 1	20.4	21.5	1.1	(−1.3 to 3.5)	0.362
	LDN × 2–3	20.3	20.7	0.4	(−1.6 to 2.4)	0.680
	LDN × 4+	20.4	19.0	−1.4	(−3.5 to 0.7)	0.184
Lamotrigine (N03AX09)	LDN × 1	4.0	4.3	0.4	(−1.4 to 2.1)	0.680
	LDN × 2–3	3.2	4.1	0.9	(−0.6 to 2.5)	0.225
	LDN × 4+	5.9	5.7	−0.2	(−1.2 to 0.8)	0.664
Gabapentin + Pregabalin (N03AX12 + N03AX16)	LDN × 1	28.8	32.4	3.6	(0.4 to 6.8)	0.026
	LDN × 2–3	28.2	30.2	2.0	(−2.2 to 6.3)	0.351
	LDN × 4+	34.8	34.2	−0.6	(−3.2 to 2.1)	0.677
Lithium (N05AM)	LDN × 1	0.5	0.5	0.0	(−0.3 to 0.2)	0.910
	LDN × 2–3	0.3	0.3	0.0	(0.0 to 0.0)	0.231
	LDN × 4+	0.7	0.6	−0.1	(−0.2 to 0.1)	0.337

Dispensed to patients one year before and after the first dispense of LDN. Three patient groups based on the number of LDN dispenses: LDN × 1 (*N* = 3590) collected LDN once, LDN × 2–3 (*N* = 2554) two or three times and LDN × 4 + (*N* = 5103) four or more times. LDN: Low dose naltrexone. DDD: Defined daily dose. ATC: Anatomical therapeutic chemical classification.

**Table 5:** Number of users of medicines in ATC subgroups (secondary outcomes).

Medicine group (ATC code)		Number of users				Difference		P
		Before		After				
		N	%	N	%	% points	95% CI	
Benzodiazepines (N05BA + N05CD + N03AE01)	LDN × 1	1072	(29.9)	1103	(30.7)	0.9	(−0.4 to 2.2)	0.189
	LDN × 2–3	672	(26.3)	704	(27.6)	1.3	(−0.3 to 2.8)	0.109
	LDN × 4+	1225	(24.0)	1209	(23.7)	−0.3	(−1.3 to 0.7)	0.539
Z hypnotics (N05CF)	LDN × 1	1168	(32.5)	1156	(32.2)	−0.3	(−1.6 to 0.9)	0.603
	LDN × 2–3	790	(30.9)	788	(30.9)	−0.1	(−1.6 to 1.4)	0.917
	LDN × 4+	1483	(29.1)	1446	(28.3)	−0.7	(−1.7 to 0.2)	0.129
Tricyclic antidepressants (N06AA)	LDN × 1	427	(11.9)	419	(11.7)	−0.2	(−1.3 to 0.9)	0.696
	LDN × 2–3	297	(11.6)	291	(11.4)	−0.2	(−1.5 to 1.0)	0.708
	LDN × 4+	590	(11.6)	469	(9.2)	−2.4	(−3.1 to −1.6)	<0.001
SSRI (N06AB)	LDN × 1	479	(13.3)	461	(12.8)	−0.5	(−1.4 to 0.4)	0.253
	LDN × 2–3	355	(13.9)	346	(13.5)	−0.4	(−1.3 to 0.6)	0.484
	LDN × 4+	603	(11.8)	566	(11.1)	−0.7	(−1.3 to −0.1)	0.016
Other antidepressants (N06AX)	LDN × 1	339	(9.4)	327	(9.1)	−0.3	(−1.2 to 0.6)	0.462
	LDN × 2–3	208	(8.1)	220	(8.6)	0.5	(−0.5 to 1.5)	0.366
	LDN × 4+	378	(7.4)	356	(7.0)	−0.4	(−1.0 to 0.2)	0.169
Lamotrigine (N03AX09)	LDN × 1	35	(1.0)	61	(1.7)	0.7	(0.3 to 1.1)	<0.001
	LDN × 2–3	28	(1.1)	32	(1.3)	0.2	(−0.3 to 0.6)	0.465
	LDN × 4+	73	(1.4)	68	(1.3)	−0.1	(−0.3 to 0.2)	0.446
Gabapentin + Pregabalin (N03AX12 + N03AX16)	LDN × 1	397	(11.1)	419	(11.7)	0.6	(−0.5 to 1.7)	0.269
	LDN × 2–3	268	(10.5)	290	(11.4)	0.9	(−0.4 to 2.2)	0.192
	LDN × 4+	562	(11.0)	470	(9.2)	−1.8	(−2.6 to −1.0)	< 0.001
Lithium (N05AM)	LDN × 1	9	(0.3)	11	(0.3)	0.1	(−0.1 to 0.2)	0.480
	LDN × 2–3	3	(0.1)	3	(0.1)	0.0	(0.0 to 0.0)	-
	LDN × 4+	16	(0,3)	12	(0,2)	−0.1	(−0.2 to 0.0)	0.046

Dispensed to patients one year before and after the first dispense of LDN. Three patient groups based on the number of LDN dispenses: LDN × 1 (*N* = 3590) collected LDN once, LDN × 2–3 (*N* = 2554) two or three times and LDN × 4 + (*N* = 5103) four or more times. LDN: Low dose naltrexone. ATC: Anatomical therapeutic chemical classification.

For all medicines being examined, there were a 6% relative increase in DDD in both LDN × 1 and LDN × 2–3, and a significant difference in-difference of 11 DDD (95% CI 1.7 to 20.3, p = 0.027) between LDN x1 and LDN × 4+. The number of users of any examined medicine showed a 6% reduction in LDN × 4+, a significant difference-in-difference compared to LDN × 1 (3.8% points, 95% CI 2.0 to 5.6, p < 0.001).

### Antiepileptics

In the LDN × 1 group, there was an 11% relative increase in the use of antiepileptics (DDD), in contrast to a non-significant reduction in the LDN × 4+ group. In number of users, there was a significant difference-in-difference between these two groups (3.1% points (95% CI 1.6 to 4.6, p < 0.001)). Significant increase in number of users was seen in both LDN x1 (+10%) and LDN × 2–3 (+14%) compared to LDN × 4+ (−11%). This is mainly attributable to changes in the dispensing of gabapentin and pregabalin, which was the dominating antiepileptics used by the included patients (73% of observed antiepileptic DDDs).

### Antipsychotics

The dispensing of antipsychotics before the index date was similar in all groups, but there was a significant 11% relative DDD increase in the LDN × 1 group, which represent a significant difference-in-difference compared to LDN × 4+ (1.4 DDD, 95% CI 0.4 to 2.3, p = 0.007). There was a 17% relative reduction in the number of antipsychotic users for the LDN × 4+ group and a 25% relative increase in LDN × 1, representing a significant difference-in-difference between these groups (2.1% points, 95% CI 1.2 to 3.0, p < 0.001).

### Benzodiazepines and Z hypnotics

There were increases in cumulative number of DDDs of benzodiazepine-like hypnotics (Z-hypnotics) in all groups, but no change in number of users. There was a significant increase in benzodiazepine DDDs for LDNx4+.

For benzodiazepines (including clonazepam), a significant increase in DDDs was seen only in LDN × 4 (+9%, relative). For all benzodiazepines and z-hypnotics combined, there was an increase in number of users in LDN × 1 (+1.3% points) compared with LDN × 4+ (−0.7% points), difference-in-difference was significant (1.9% points (95% CI 0.2 to 3.7, p = 0.035)).

### Antidepressants

There were no significant differences in DDDs of antidepressants in any group. For the number of users of antidepressants, there was a significantly larger reduction in LDN × 4+ (−21%, relative) than in LDN × 1 (−2%), which also constituted a significant difference-in-difference (2.8% points, 95% CI 1.1 to 4.4, p = 0.001).

For LDN × 4+, there were significant reductions in DDD in tricyclic antidepressants (−8% relative), and in the number of users of tricyclic antidepressants (−21%) and SSRI (−6%). There was difference-in-difference in number of users (2.1% points, 95% CI 0.8 to 3.5, p = 0.003) of tricyclic antidepressants between LDN × 1 (−0.2% points) and LDN × 4 + (−2.4% points).

### Psychostimulants

There were no changes in the dispensing of psychostimulants following the initiation of LDN use.

### Mood stabilizers

There was a 13% relative increase in the number of DDDs for gabapentin and pregabalin in the LDN × 1 group, but no difference in cumulative sum of DDDs for lithium and lamotrigine in any group. There was a relative 70% increase in the number of lamotrigine users in LDN x1 and a significant difference-in-difference from LDN × 4+ (0.8% points (95% CI 0.4 to 1.3, p = 0.001).

For gabapentin and pregabalin, there was also a significant difference-in-difference in the number of users (2.4% points, 95% CI 1.0 to 3.8, p = 0.001) between. LDN × 1 (+0.6% points) and LDN × 4+ (−1.8% points).

## Discussion

We have shown that starting LDN was followed by changes in the dispensing of several psychotropic medicines. When comparing persistent LDN users (LDN × 4+) with individuals who collected LDN only once (LDN x1), there were significant difference-in-differences in DDDs of antipsychotics, and in the number of users of antiepileptics, antipsychotics, antidepressants, tricyclic antidepressants, lamotrigine and gabapentin and pregabalin. In all of these groups, the reductions in number of users were larger in LDN × 4+. There was a dose-response relationship between reduction in number of users and increasing LDN exposure for antiepileptics, antipsychotics and antidepressants.

Major strengths of this study are the number of included participants, the completeness of the data, and the extended observation period. The number of participants was larger than in any other study on LDN, and the NorPD is a reliable, comprehensive, and complete register of all dispenses of prescription medicines to all Norwegian residents. The observations are from a real-world setting, and should be relevant for how LDN affect the use of other medicines at a population level.

Before-after studies have some limitations. Participants are not randomly recruited or assigned to groups, and there is risk of both inclusion and assignment bias. The differences in sex and age were minor, but as there were major differences in the dispensing of medicines before starting LDN (e.g. anxiolytics) that may indicate differences in psychiatric morbidity between groups. We still believe the analyses are valuable since before-after comparisons were made both within groups and between groups. By choosing differences in DDDs and number of users as main outcome measures, and also analyzing for difference-in-difference between groups also increase the generalizability of the study. Ideally, a control group unexposed to LDN should have been included, but our ethics data privacy approvals did not allow this.

Quasi-experimental studies are prone to deviation to a mean, and examples were present in this study (e.g. antiepileptic DDD)^19^. However, there were also several examples that the results deviated away from the pre-LDN mean of the total study population.

The analyses include multiple comparisons, and the findings should, therefore be interpreted with caution. It is likely some significant results found were caused by chance. However, the magnitude of some of the findings and the presence of dose-effect relationships suggest other explanations than chance alone.

NorPD records all dispenses outside hospitals and institutions. Dispenses to hospitalized patients could potentially lead to bias. We believe this is negligible since most LDN was prescribed in general practice. Less than 1% of LDN prescribers in Norway were psychiatrists^10^.

It is also important to remind that the NorPD contains very little clinical information. We focused on the dispensing of medicines, not indications for use or clinical condition. Assessment of compliance was impossible.

In a previous study, we showed that opioid use was reduced by 47% after starting LDN. This was not compensated by increased use of NSAID or other non-opioid analgesics^13^. In this study, we find that the number of users of adjuvant pain medication, such as gabapentin, pregabalin and tricyclic antidepressants, was reduced in persistent LDN users. This further substantiates assumptions that LDN may have therapeutic benefits in chronic pain conditions. However, it is possible that quitting opioids could be a part of the observed effects on psychotropic medicines more than actual therapeutic effects.

We did not observe any reduction in the dispensing of anxiolytics or hypnotics. On the contrary, there were increases in the cumulative dose for z-hypnotics for all groups. Sleep disturbances are among the most frequently reported adverse effects of LDN, and may contribute together with opioid withdrawal to an increased need for hypnotics.

One explanation to our findings is that patients experience beneficial effects that lead to reduced need for psychotropic drugs. It is plausible that naltrexone could influence mental health by interfering with the endogenous opioids, which may have a direct role in depression via the mesolimbic system^20^. Indications of a relationship between inflammation and psychiatric conditions such as depression^21^ and schizophrenia^22^ have paved the way for a separate field of research: Psychoneuroimmunology. The suggested immunomodulary effects of LDN could have implications for psychiatry.

In conclusion, the reductions in the use of antiepileptics and psychotropics following the initiation of persistent LDN use suggest that efficacy of LDN in depression and other psychiatric conditions cannot be ruled out. Due to the limitations of this pharmacoepidemiological study, the results should not directly lead to changes in clinical practice. Randomized clinical studies on LDN in psychiatry should be conducted. LDN could be an efficacious, safe and affordable treatment alternative.

## Data Availability

Data is available from the Norwegian Prescription Database (NorPD) for researchers who after application meet the criteria for access to prescription data. This includes Ethics Committee evaluation and a fee will be charged. For further details, please see https://www.fhi.no/en/op/data-access-from-health-registries-health-studies-andbiobanks/norwegian-prescription-database/Access-data-norpd/. We did not have any special privileges accessing the data, we obtained the data through the same application and fee payment process as outlined here. Our approvals do not allow the publication of raw data used in this study. In accordance with NorPD’s requirements, we had to delete all original data by 31 December 2018.
